# Calciphylaxis

**Published:** 2015-02-19

**Authors:** Jennifer Harb, Anthony W. Watt, Joshua B. Elston, Michael A. Harrington

**Affiliations:** Division of Plastic Surgery, Department of Surgery, University of South Florida Morsani College of Medicine, Tampa, Fla

**Keywords:** calcific uremic arteriolopathy, calciphylaxis, cutaneous ulcerations, ESRD, sepsis

## DESCRIPTION

A 56-year-old man presented with a wound of the right leg for 6 weeks that progressed from a linear ulceration with surrounding erythema to an excruciatingly painful ulcer with fat saponification and visible thrombosed vessels. His medical history was significant for dialysis-dependent kidney failure for the past 14 years.

## QUESTIONS

1. **How is calciphylaxis diagnosed?**
2. **What are the risk factors?**
3. **What is the pathophysiology?**
4. **What are management options, and what role does wound care play?**

## DISCUSSION

Calciphylaxis, otherwise known as calcific uremic arteriolopathy (CUA), is a diagnosis largely based on clinical findings in the setting of kidney failure. Lesions begin as tender, violaceous plaques and nodules that quickly progress to excruciatingly painful necrotic ulcerations.^1^ These typically follow the underlying vessels in a linear pattern and can be differentiated from peripheral vascular disease by an intact peripheral pulse.^2^ The lower extremity is most commonly involved (92%), followed by the trunk (30%).^3^ X-ray may show intimal and arborizing calcification of vessels. Diagnostic tissue confirmation requires deep skin biopsy (not punch) with subcutaneous adipose tissue. The most common histological finding is metastatic calcification of dermal and subcutaneous arterioles and calcifying septal panniculitis. A unique feature is the phenotypic differentiation of smooth muscle cells into osteoid-like cells.^1^^,^^4^^-^6 The differential diagnosis should rule out other processes such as venous stasis ulcers, pyoderma gangrenosum, vasculitis, brown recluse spider bite, and necrotizing fasciitis. Delay in diagnosis may be common; however, this has not been shown to affect survival.^1^

Important risk factors for development of CUA are kidney failure, female sex (5:1 predominance), obesity, corticosteroid use, and liver disease.^3^ The pathophysiology of CUA is not fully understood, but it is ultimately related to excess serum calcium and phosphate that lead to a calcifying arteriolopathy. The metastatic extraosseous calcifications result from the excess serum calcium phosphate complexes in renal failure that exceed their solubility leading to passive mineralization and deposition as arteriolar crystals.^4^ Direct endothelial injury from calcification and decreased blood flow promote thrombosis with dermal and subcutaneous ischemic necrosis.^5^

There is no consensus algorithm for treatment of calciphylaxis; however, it is clear that the approach must be multimodal. Primary focus should be placed on aggressively reducing serum calcium phosphate complexes with bisphosphonates and cinacalcet to prevent worsening of the disease.^2^^,^^4^^-^6 More recently, sodium thiosulfate has been used as a symptomatic adjunct to reduce pain and the presence of ulcerations despite no improvement in survival.^2^^,^^5^^-^7 Recent retrospective studies have shown that operative debridement can increase survivability with 1 study demonstrating improved 1-year survivability with debridement versus no debridement (61.6% vs 27.4%, respectively).^3^ At this time, the literature would suggest debridement is paramount as the necrotic ulcers could result in wound infection, gangrene, amputation, and sepsis—the most feared complication. Aggressive wound care with a broad-spectrum agent (silver sulfadiazine or moistened Silverlon gauze) is critical to prevent superinfection of open areas. The role of parathyroidectomy is debated due to the inability to reproduce decreased mortality rates.^5^ Finally, pain management consults are frequently warranted. Overall response to any treatment regimen is poor with a 1-year survival rate of only 45.8%.^3^

Plastic surgeons are often consulted for difficult wounds. Accurate diagnosis and identification of the wound etiology is critical in providing a successful plan of care. Calciphylaxis is a rare and devastating form of microvascular occlusion syndrome leading to progressive skin necrosis. Lesions rapidly progress to exquisitely painful ischemic ulcerations most commonly involving the legs and trunk.^3^ It is important to recognize the pattern of scattered and rapidly progressive thrombotic vaso-occlusive ulcers in the setting of longstanding and often dialysis-dependent renal failure. Debridement has been shown to improve survivability and aggressive broad-spectrum topical wound care should be initiated. Ultimately, the cutaneous manifestations of calciphylaxis are often the heralding signs of a chronic syndrome that portends a grim prognosis.

## Figures and Tables

**Figure 1:**
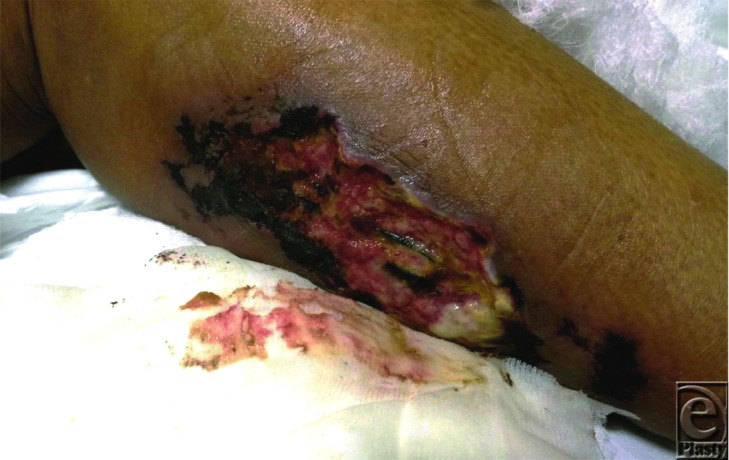
Right leg with painful ulcer demonstrating thrombosed blood vessels, saponified fat, and ischemic skin necrosis.
